# G protein-coupled estrogen receptor mediates anti-inflammatory action in Crohn’s disease

**DOI:** 10.1038/s41598-019-43233-3

**Published:** 2019-05-01

**Authors:** Damian Jacenik, Marta Zielińska, Anna Mokrowiecka, Sylwia Michlewska, Ewa Małecka-Panas, Radzisław Kordek, Jakub Fichna, Wanda M. Krajewska

**Affiliations:** 1 Department of Cytobiochemistry, Faculty of Biology and Environmental Protection, University of Lodz, Pomorska St. 141/143, 90-236 Lodz, Poland; 2 Department of Biochemistry, Faculty of Medicine, Medical University of Lodz, Mazowiecka St. 6/8, 92-215 Lodz, Poland; 3 Department of Digestive Tract Diseases, Faculty of Medicine, Medical University of Lodz, Stefana Kopcinskiego St. 22, 90-001 Lodz, Poland; 4 Department of General Biophysics, Faculty of Biology and Environmental Protection, University of Lodz, Pomorska St. 141/143, 90-236 Lodz, Poland; 5 Laboratory of Microscopic Imaging and Specialized Biological Techniques, Faculty of Biology and Environmental Protection, University of Lodz, Banacha St. 12/16, 90-237 Lodz, Poland; 6 Department of Pathology, Chair of Oncology, Medical University of Lodz, Pomorska St. 521, 92-213 Lodz, Poland

**Keywords:** Steroid hormones, Crohn's disease

## Abstract

Estrogens exert immunomodulatory action in many autoimmune diseases. Accumulating evidence highlights the meaningful impact of estrogen receptors in physiology and pathophysiology of the colon. However, the significance of G protein-coupled estrogen receptor (GPER) on Crohn’s disease (CD), one of the inflammatory bowel disease (IBD) types, is still elusive. Our study revealed GPER overexpression at the mRNA and protein levels in patients with CD. To evaluate the effects of GPER activation/inhibition on colitis development, a murine 2, 4, 6-trinitrobenzene sulfonic acid (TNBS)-induced model of CD was used. We showed that activation of GPER reduces mortality, improves macroscopic and microscopic scores and lowers C-reactive protein (CRP) level. The impact of estrogen signaling on the suppression of the intestinal inflammation was proved by immunohistochemistry. It was demonstrated that GPER activation is accompanied by modulation of extracellular-signal regulated kinase (ERK) signaling pathway and expression level of genes involved in signal transmission and immune response as well as the expression of some microRNAs (miR-145, miR-148-5p and miR-592). Our study revealed that the membrane-bound estrogen receptor GPER mediates anti-inflammatory action and seems to be a potent therapeutic target in maintaining remission in CD.

## Introduction

Crohn’s disease (CD) is one of the inflammatory bowel disease (IBD) types characterized by chronic intestine inflammation. Although intestinal microbiota has been identified as the main factor responsible for immune response alternation in the past decade, other factors involved in CD pathogenesis, such as genetic and environmental, have been described. Etiology of CD is still poorly understood and treatment of CD patients generally consists of maintaining long-term remission. There are some therapeutic strategies but unfortunately many of them appear to be not effective in all hospitalized CD patients. For instance, it was shown that over a half of CD patients who received corticosteroid therapy did not respond to treatment or needed surgery intervention^1^. Consequently, biological therapy such as anti-tumor necrosis factor α (TNFα) or anti-α4-integrin is the most effective approach for CD patients^2^. Nevertheless, some studies indicate that about 40% of IBD patients have been identified as non-responding to treatment and many of anti-TNFα-treated patients are developing resistance for anti-TNFα monoclonal antibody^3^.

It has been documented that estrogens can regulate chronic inflammatory diseases such as arthritis, systematic lupus erythematosus, experimental autoimmune encephalomyelitis, thyroiditis, endometriosis and colon inflammation^4^. Epidemiological studies indicate that oral contraceptives (OC) and hormone replacement therapy (HRT) in post-menopausal women are related with occurrence and development of IBD. Kane *et al*. documented that hormone replacement therapy in post-menopausal women with either IBD type, i.e. CD and ulcerative colitis (UC) is related with reduction of disease activity as compared to non-HRT users^5^. Additionally, decline of IBD activity in post-menopausal women seems to be associated with duration of exogenous hormone supplementation. On the other hand, it was estimated that OC or HRT current users have increased risk of developing CD^6^.

The effect of estrogens is mediated by interaction of estrogens with cognate receptors and triggering estrogen-dependent signaling pathways. G protein-coupled estrogen receptor (GPER, earlier known as GPR30) is a membrane-bound estrogen receptor responsible for non-genomic action of estrogens. In contrast to nuclear estrogen receptors, i.e. ERα and ERβ which are liable to direct gene regulation, activation of GPER results in rapid modulation of several proteins, leading to signal transduction and changes at the transcriptional level. GPER is widely distributed in human tissues including intestine where it may act as a potent mediator of the immune response. Studies of metabolic syndrome in GPER knockout mice revealed higher level of plasma pro-inflammatory cytokines compared to wild type mice^7^. In line, it has been documented that GPER exerts an anti-inflammatory effect in endothelium and vascular tissue^8,9^.

However, so far only limited efforts have been made to learn about the role of GPER in IBD. It is suggested that GPER which expression has been found to be elevated in CD may be a potent mediator of immune response^10^. The aim of our studies was to clarify the impact of GPER activation and inhibition on colon inflammation in the context of estrogen and other signaling pathways and genes associated with the immune response. The research was carried out using the 2, 4, 6-trinitrobenzene sulfonic acid (TNBS)-induced murine model of CD. Effects of GPER specific agonist G-1 and antagonist G-15 as well as 17β-estradiol and ICI 182.780 (Fulvestrant^®^ or Faslodex^® ^) which is the antagonist for nuclear estrogen receptors but agonist for GPCR were analyzed.

## Results

### GPER is overexpressed in patients with Crohn’s disease

**Figure 1:**
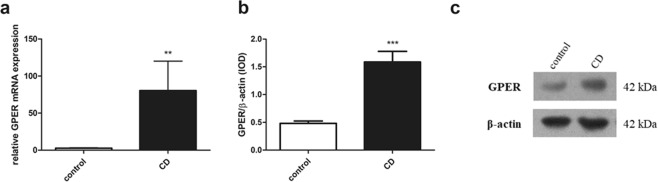
GPER expression at the mRNA (**a**) and protein level (**b**) in men with CD. Representative immunoblot of GPER protein analysis (**c**). Data are presented as means ± SEM; **P < 0.01, ***P < 0.001 CD vs. control.

We analyzed local expression of GPER at the mRNA and protein level in intestinal mucosa samples obtained from men with CD and sex and age-matched control group. In men with CD, real-time PCR analysis showed over 29-fold (*P* < 0.01) increase of GPER mRNA expression level (Fig. 1a). Western blot analysis confirmed up-regulation of GPER protein level in men with CD in relation to control group (Fig. 1b,c; see Supplementary Fig. S1). About 3-fold (*P* < 0.001) higher GPER protein level in men with CD was noted.

### GPER activation lead to improvement of macroscopic characteristics of colon and reduces mortality in murine model of Crohn’s disease

**Figure 2:**
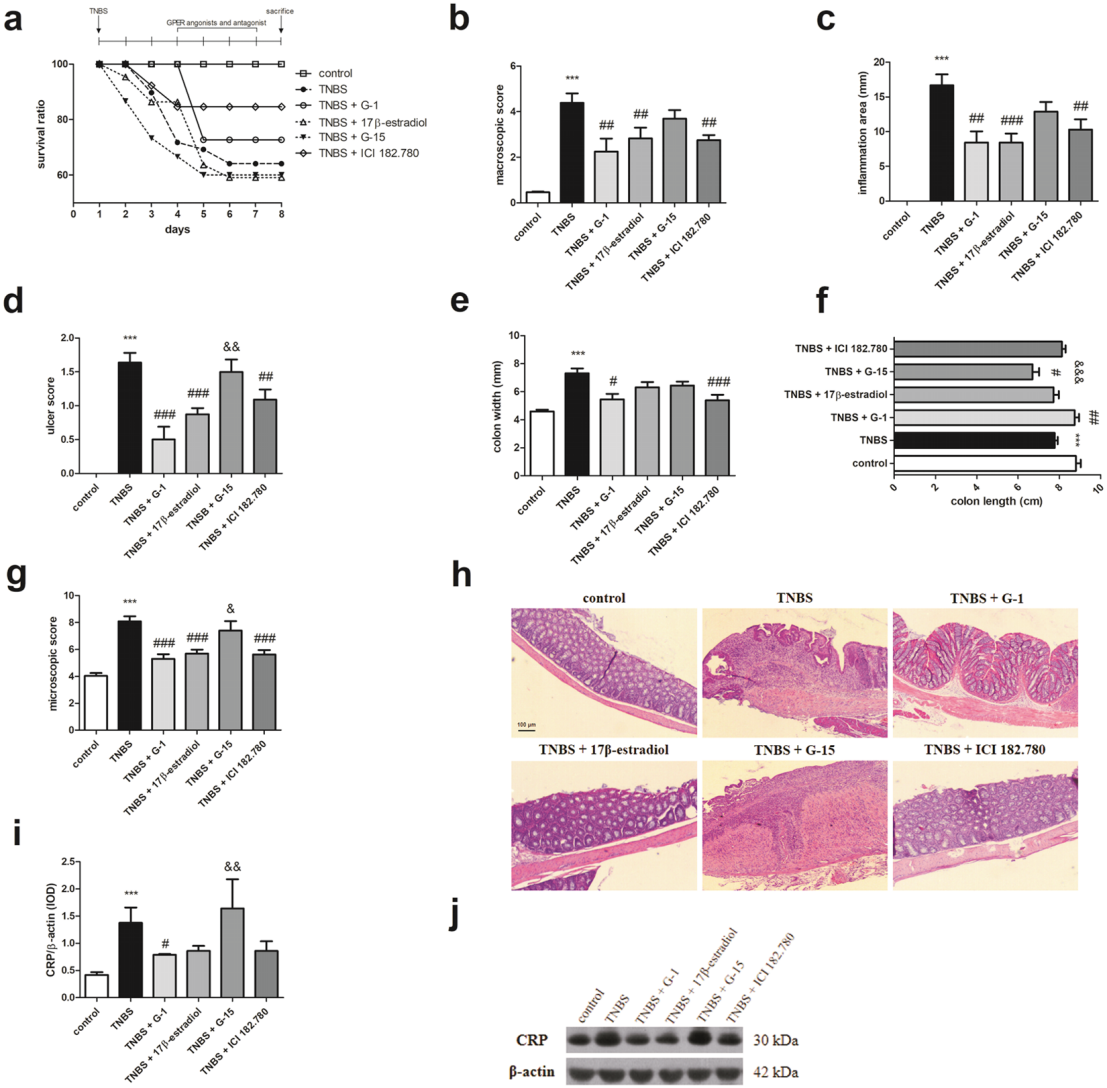
Survival ratio (**a**), macroscopic score (**b**), inflammation area (**c**), ulcer score (**d**), colon width (**e**), colon length (**f**) and microscopic score (**g**) in control, TNBS and G-1, 17β-estradiol, G-15 and ICI 182.780 treated groups. Representative images of hematoxylin and eosin staining of colon cross-section obtained from control, TNBS and treated groups – type of treatment over each images is shown. CRP (**i**) protein level and representative immunoblot of CRP protein analysis (**j**) in colon section obtained from control, TNBS and treated groups. Data are presented as means ± SEM; ***P < 0.001 TNBS vs. control; ^#^*P* < 0.05, ^##^*P* < 0.01, ^###^*P* < 0.001 treated groups vs. TNBS; ^&^*P* < 0.05, ^&&^*P* < 0.01, ^&&&^*P* < 0.001 G-15 treated group vs. G-1 treated group. 10–15 mice per group. Ten independent cross-sections per group were examined. Scale bars, 100 µm. Representative blots were cropped from the same gels.

Induction of colon inflammation by TNBS administration significantly increased macroscopic score (*P* < 0.001), inflammation area (*P* < 0.001), ulcer score (*P* < 0.001) and colon width (*P* < 0.001) when compared with control (Fig. 2b–e). In line, statistically significant smothering of colon length in TNBS group (*P* < 0.001) in relation to control group was observed (Fig. 2f). To evaluate the significance of GPER as a mediator of colon inflammation we analyzed impact of GPER activation and inhibition on CD in murine model. Macroscopic analysis of colon fragments taken from each studied group proved that G-1 (*P* < 0.01), 17β-estradiol (*P* < 0.01) or ICI 182.780 (*P* < 0.01) administration was associated with improvement of macroscopic parameters (Fig. 2b). Statistically significant reduction of inflammation area, ulcer score and colon width after GPER activation was demonstrated (Fig. 2c–e). Myeloperoxidase (MPO) activity level was tended to be up-regulated in TNBS group in relation to control group, while down-regulation was observed after the G-1 or ICI 182.780 treatment (see Supplementary Fig. S2). Our analysis also showed GPER-dependent changes in colon length in murine model of CD (Fig. 2f). It is noteworthy that the differences in macroscopic and microscopic effects induced by G-1 compared to G-15 are statistically significant. Simultaneously, survival ratio analysis indicated that selective activation of GPER *via* specific agonists G-1 and ICI 182.780 administration led to a decrease in mortality (Fig. 2a). However, GPER inhibition *via* selective antagonist G-15 supplementation did not affect survival ratio in relation to TNBS group (Fig. 2a).

### GPER activation lead to improvement of microscopic score in murine model of Crohn’s disease

Based on histological analysis we evaluated effect of GPER modulation on colon architecture. Colon samples obtained from TNBS group were characterized by higher microscopic damage score (Fig. 2g,h). Granuloma, immune cell infiltration and ulceration were observed in semi-chronic colon inflammation and in case of GPER inhibition by G-15 administration in our murine model of CD (Fig. 2g,h). Contrarily, G-1 (*P* < 0.001), along with 17β-estradiol (*P* < 0.001) or ICI 182.780 (*P* < 0.001) administration led to improvement of colon architecture. Involvement of GPER in immune response in colon was confirmed by C-reactive protein (CRP) level evaluation. Higher CRP level (*P* < 0.001) in TNBS group in relation to control group was observed (Fig. 2i,j). GPER activation resulted in decline of CRP protein level but only in the case of G-1 (*P* < 0.05) the decrease was statistically significant (Fig. 2i; see Supplementary Fig. S3).

### Lower GPER expression is related with decline of colon inflammation in murine model of Crohn’s disease

**Figure 3:**
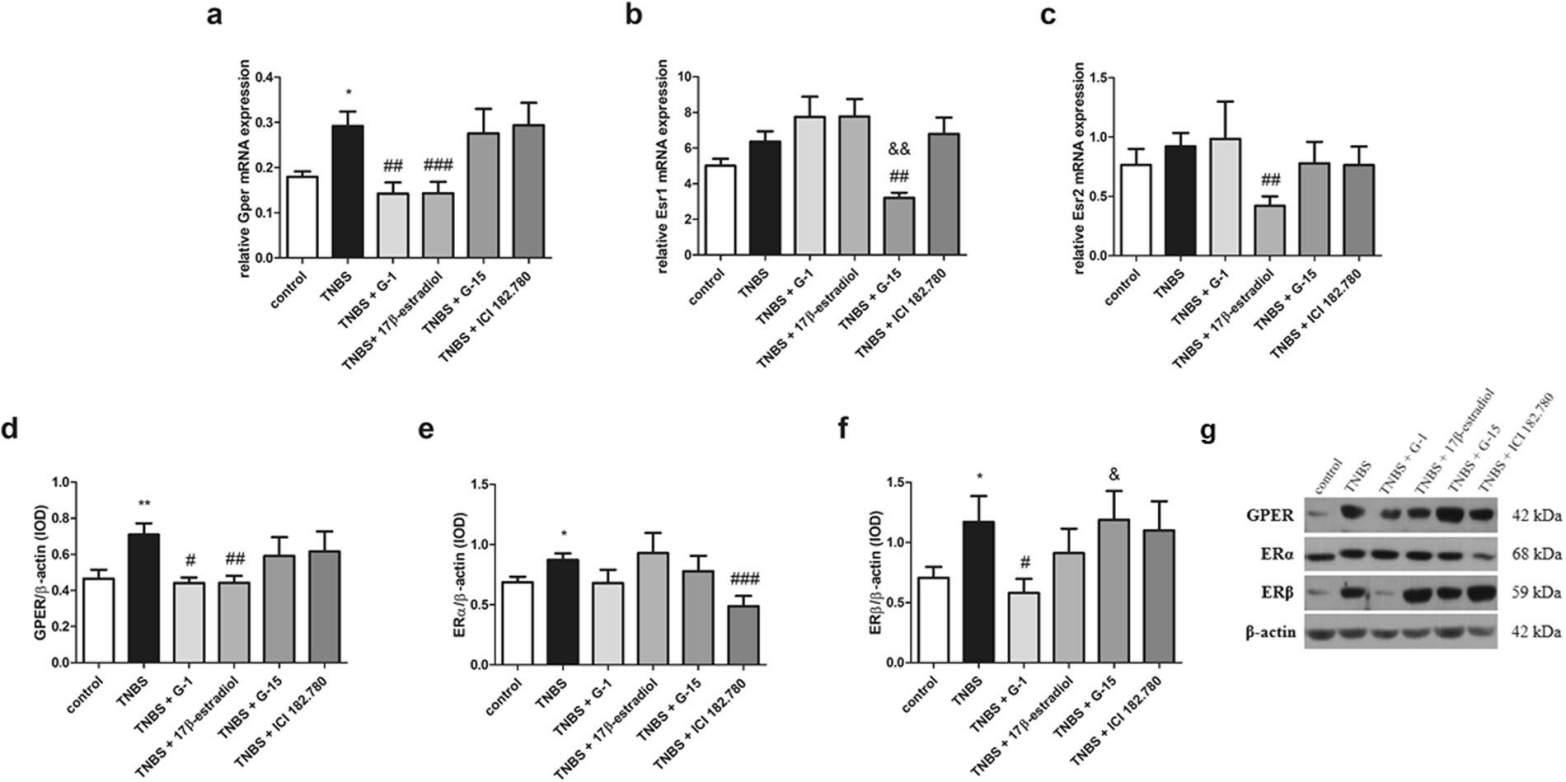
Gper/GPER (**a**,**d**), Esr1/ERα (**b**,**e**) and Esr2/ERβ (**c**,**f**) expression at the mRNA (**a**–**c**) and protein level (**d**–**f**) and representative immunoblot of GPER, ERα and ERβ protein analysis (**g**) in colon section obtained from control, TNBS and treated groups. Data are presented as means ± SEM; *P < 0.05, **P < 0.01 TNBS vs. control; ^#^*P* < 0.05, ^##^*P* < 0.01, ^###^*P* < 0.001 treated groups vs. TNBS; ^&^*P* < 0.05, ^&&^*P* < 0.01 G-15 treated group vs. G-1 treated group. 10–15 mice per group. Representative blots were cropped from the same gels.

In our murine model of CD an increased expression of GPER at the mRNA (*P* < 0.05) and protein (*P* < 0.01) level was demonstrated (Fig. 3a,d,g; see Supplementary Fig. S4). Decreased expression of GPER at the mRNA level after G-1 (*P* < 0.01) and 17β-estradiol (*P* < 0.001) treatment was observed (Fig. 3a). In line, decline of GPER at the protein level in the presence of G-1 (*P* < 0.05) and 17β-estradiol (*P* < 0.01) was noted (Fig. 3d,g). Our analysis revealed up-regulation of ERα (*P* < 0.05) and ERβ (*P* < 0.05) expression, but only in the case of protein changes between control and TNBS groups were statistically significant (Fig. 3b,c,e,f,g; see Supplementary Fig. S4). Interestingly, in the presence of G-15, a selective antagonist of GPER, down-regulation of ERα mRNA expression (*P* < 0.01) was stated. When we analyzed protein content, we observed statistically significant lower ERα protein level in murine model of CD after treatment with ICI 182.780 (*P* < 0.001) (Fig. 3b,e,g). In the case of ERβ, 17β-estradiol led to a decrease of ERβ mRNA expression (*P* < 0.01) (Fig. 3c) while G-1 treatment resulted in down-regulation of ERβ at the protein level (*P* < 0.05) in relation to TNBS group (Fig. 3f,g).

### Differences in estrogen receptor localization in murine model of Crohn’s disease

**Figure 4:**
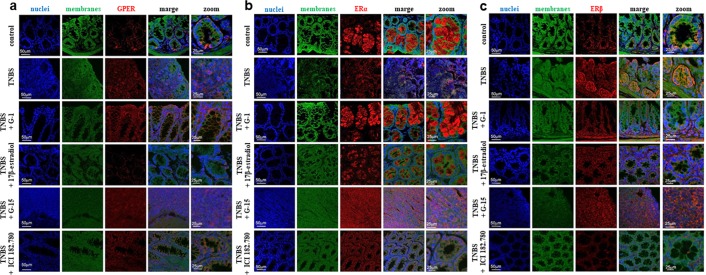
Representative images of immunohistochemical staining of GPER (**a**), ERα (**b**) and ERβ (**c**) in colon cross-section obtained from control, TNBS and treated groups – type of treatment on the left side of each images is shown. 10–15 mice per group. Ten independent cross-sections per group were examined. Scale bars, 50 µm; zoom scale bars, 25 µm.

Immunohistochemistry (IHC) analysis revealed predominantly cytoplasmic localization of GPER in a cross-section obtained from our murine model of CD (Fig. 4a). Strong cytoplasmic staining of GPER in enterocytes and membrane of goblet cells in G-1 and ICI 182.780-treated groups was found. GPER cytoplasmic staining in the apical domain of the crypts in the case of 17β-estradiol-treated murine model of CD was noted. Moreover, both nuclei and cytoplasmic staining after inhibition of GPER was observed. Our confocal microscopic study showed that ERα is localized solely in cytoplasm of goblet cells after activation by specific GPER agonist G-1 and 17β-estradiol. On the other hand, in the TNBS group which was supplemented with G-15, strong nuclei staining of ERα protein was noted (Fig. 4b). Strong cytoplasmic staining of ERβ in the apical and subapical domain of the crypts and epithelium in cross-sections obtained from the TNBS group was documented. Similarly, ERβ was localized in cytoplasm in mucosa and also in submucosa in G-1-treated group. 17β-estradiol-treated group was characterized by localization of ERβ in crypts, cytoplasm and membrane of goblet cells. Cytoplasmic staining of ERβ in both G-15 and ICI 182.780-treated group was observed (Fig. 4c). Interestingly, our IHC study revealed lack of GPER and ERα or ERβ as well as ERα and ERβ co-localization in our murine model of CD and treated groups (data not shown).

### GPER mediates ERK signaling in murine model of Crohn’s disease

**Figure 5:**
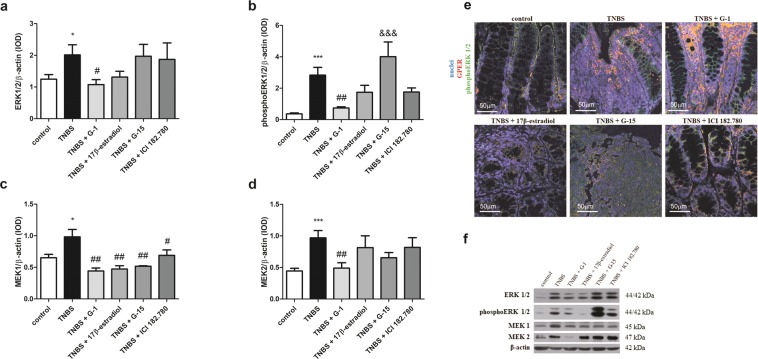
ERK 1/2 (**a**), phosphoERK 1/2 (**b**), MEK 1 (**c**) and MEK 2 (**d**) protein level and representative immunoblot of ERK 1/2, phosphoERK 1/2, MEK 1 and MEK 2 protein analysis (**f**) in colon section obtained from control, TNBS and treated groups. Representative images of immunohistochemical staining of GPER and phosphoERK 1/2 (**e**) in colon cross-section obtained from control, TNBS and treated groups – type of treatment over each images are shown. Data are presented as means ± SEM; *P < 0.05, ***P < 0.001 TNBS vs. control; ^#^*P* < 0.05, ^##^*P* < 0.01 treated groups vs. TNBS; ^&&&^*P* < 0.001 G-15 treated group vs. G-1 treated group. 10–15 mice per group. Ten independent cross-section per group were examined. Scale bars, 50 µm. Representative blots were cropped from the same gels.

Up-regulation of extracellular signal-regulated kinase (ERK) 1/2 (*P* < 0.05) and phosphoERK 1/2 (*P* < 0.001) at the protein level in CD murine model in relation to control group was noted (Fig. 5a,b; see Supplementary Fig. S5). Western blot analyses indicated that G-1 administration results in lowering of ERK 1/2 (*P* < 0.05) protein level in relation to TNBS group (Fig. 5a,f). In line, GPER activation leads to down-regulation of phosphoERK 1/2 at the protein level but only in the case of G-1 treatment (*P* < 0.01) the decrease was statistically significant (Fig. 5b,f). In contrast, no differences in AKT and phosphoAKT kinase protein level in studied groups were found (see Supplementary Fig. S6 and S7). Immunohistochemistry analysis revealed co-localization of GPER and phosphoERK 1/2 mainly in enterocytes (Fig. 5e). Interestingly, stronger GPER and phosphoERK 1/2 staining in the case of the TNBS group supplemented with the GPER selective agonist G-1 was observed (Fig. 5e). To confirm cross-talk between GPER and ERK signaling we analyzed mitogen-activated protein kinase kinase (MEK) 1 and 2 in our murine model of CD. Higher MEK 1 (*P* < 0.05) and MEK 2 (*P* < 0.001) protein content in murine CD samples compared to control samples was stated (Fig. 5c,d; see Supplementary Fig. S5). Down-regulation of MEK 1 at the protein level was demonstrated after G-1 (*P* < 0.01), 17β-estradiol (*P* < 0.01), G-15 (*P* < 0.01) and ICI 182.780 (*P* < 0.05) treatment compared to the TNBS group (Fig. 5d,f). Statistically significant decrease of MEK 2 expression after G-1 supplementation (*P* < 0.01) in relation to TNBS group was also found (Fig. 5e,f).

### GPER mediates expression of signal transmission and immunomodulatory genes as well as selected microRNA in murine model of Crohn’s disease

**Figure 6:**
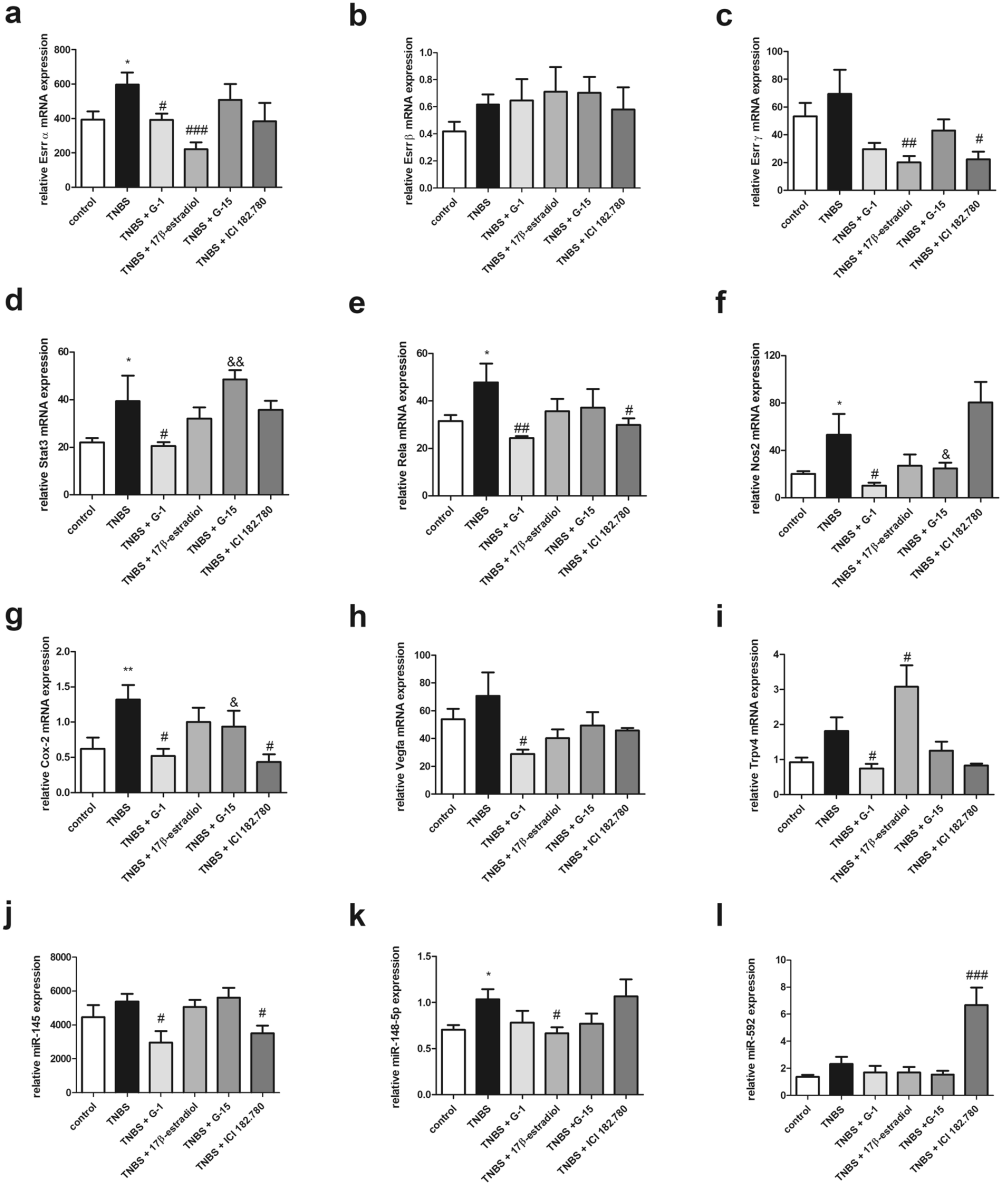
Expression at the mRNA level of genes involved in signal transmission and immune response as well as selected microRNA in colon section obtained from control, TNBS and treated groups. Data are presented as means ± SEM; *P < 0.05, **P < 0.01 TNBS vs. control; ^#^*P* < 0.05, ^##^*P* < 0.01, ^###^*P* < 0.001 treated groups vs. TNBS; ^&^*P* < 0.05, ^&&^*P* < 0.01 G-15 treated group vs. G-1 treated group. 10–15 mice per group.

Lower estrogen-related receptor (Esrr) α and γ mRNA expression levels were disclosed in colon samples obtained from G-1 (*P* < 0.05) or 17β-estradiol treated (*P* < 0.001) and from 17β-estradiol (*P* < 0.01) or ICI 182.780 (*P* < 0.05) treated groups, respectively in relation to the TNBS group (Fig. 6a,c). It is worth to note that statistically significant overexpression of Esrrα (*P* < 0.05) in TNBS group in relation to control group was observed (Fig. 6a). No differences in Esrrβ mRNA expression level between the TNBS group and treated groups were noted (Fig. 6b). What is important, modulatory effects of GPER activation and inhibition on signal transducer and activator of transcription 3 (Stat3) mRNA expression level in TNBS model of CD was documented. Lower mRNA copy number of Stat3 (*P* < 0.05) after GPER activation by G-1 administration in our murine model of CD was indicated. In turn, GPER inhibition by G-15 supplementation increases Stat3 mRNA (Fig. 6d). It was estimated that CD induction in mice leads to overexpression of nuclear factor NFκB subunit (Rela) mRNA (*P* < 0.05). Activation of GPER *via* G-1 (*P* < 0.01) and ICI 182.780 (*P* < 0.05) causes down-regulation of Rela expression at the mRNA level in relation to the TNBS group (Fig. 6e). Our real-time PCR analysis documented overexpression of inflammatory markers such as nitric oxide synthase 2 (Nos2) (*P* < 0.05) and cycloxyganes-2 (Cox-2) (*P* < 0.05) in TNBS group compared to control group (Fig. 6f,g). Statistically significant lower level of Nos2 mRNA expression in colon samples taken from G-1 treated mice with CD (*P* < 0.05) in relation to TNBS group was shown (Fig. 6f). Specific GPER activation by G-1 (*P* < 0.05) and ICI 182.780 (*P* < 0.05) results also in down-regulation of Cox-2 mRNA (Fig. 6g). Despite the lack of statistical significance in the case of vascular endothelial growth factor (Vegfa) and transit receptor potential vanilloid 4 (Trpv4) mRNA analysis, we observed increasing trend of Vegfa and Trpv4 mRNA expression in murine model of CD in relation to control group (Fig. 6h,i). Additionally, decline of Vegfa mRNA level associated with G-1 treatment in murine model of CD was documented (Fig. 6h). TNBS mice treated with G-1 were characterized by lower Trpv4 mRNA expression (*P* < 0.05) in contrast to 17β-estradiol which was able to enhance copy number of Trpv4 (*P* < 0.05) in relation to the TNBS group (Fig. 6i). In murine model of CD, higher miR-145, miR-148-5p and miR-592 expression compared to control group was noted but only in the case of miR-148-5p this change was statistically significant (*P* < 0.05). Selective activation of GPER by G-1 (*P* < 0.05) and ICI 182.780 (*P* < 0.05) was found to be associated with down-regulation of miR-145 copy number in TNBS model (Fig. 6j). Lower expression of miR-148-5p was documented after 17β-estradiol treatment (*P* < 0.05) while ICI 182.780 supplementation has been shown to be accompanied by overexpression of miR-592 (*P* < 0.001) in our murine model of CD (Fig. 6k,l).

## Discussion

Previous studies showed that hormone fluctuation during menses and pregnancy are associated with changes in IBD symptoms^11^. Moreover, modulatory effect of female hormones on pre-menstrual and menstrual syndrome such as irritability, depression, pelvic pain or diarrhea during menstrual cycle in IBD is well documented^12,13^. Of note, experimental evidence indicated that estrogens may also be involved in pathophysiology of some male-related diseases such as prostate cancer^14^. Bábíčková *et al*. documented more significant colon shortening, worse stole score, stronger histological injury and higher TNFα level in colon samples obtained from male mice compared to female mice with colitis^15^. Anti-inflammatory action caused by 17β-estradiol treatment such as down-regulation of pro-inflammatory cytokines in TNBS-induced acute colitis in ovariectomized female mice was demonstrated. The effect of estrogen treatment in acute colitis was found to be dependent on the expression status of ERβ^16^.

Accumulating evidence suggests that nuclear estrogen receptors are involved in pathogenesis of IBD. Harnish *et al*. found some nuclear estrogen receptor-mediated beneficial effect of estrogen in the HLA-B27 transgenic rat model of IBD^17^. Lower level of ERβ in intestinal mucosa and peripheral T cells obtained from CD patients, IBD patients in endoscopic activity and IBD patients unresponsive for anti-TNFα therapy was documented. Interestingly, *in vitro* experiments revealed that interleukin (IL)-6 is able to influence ERβ expression in T cells and intestinal mucosa^18^. On the other hand, the increased expression of ERα in peripheral T cells obtained from CD patients was noted^18^. It was also shown that ERα deletion in T cells protects against colitis development. ERα seems to take part in activation, proliferation and differentiation of T cells^19^. It has also been demonstrated that low ratio of circulating ERβ and ERα is associated with clinical and endoscopic activity of CD and seems to be useful to non-invasive disease activity monitoring in CD patients^20^. Thus estrogens seem to play crucial role in IBD. However, it remains unclear whether estrogen membrane-bound estrogen receptor, i.e. GPER is associated with CD pathophysiology.

In our study we showed that intestinal mucosa samples obtained from patients with CD are characterized by overexpression of GPER. Evidence suggesting impact of GPER in IBD has been provided by Włodarczyk *et al*., who observed significant differences in GPER protein level between inflamed and non-inflamed CD tissues, regardless of sex^10^. Therefore, we postulate that GPER may be one of the crucial mediators of the immune response in CD. Presented study was carried out on a CD model induced in male mice to exclude the possibility that effects evaluated in our experiments may be the result of potential changes in estrogen levels depending on sex or age. We demonstrated that specific GPER activation mediates anti-inflammatory action in murine model of CD. Macroscopic evaluation revealed GPER-mediated effect on macroscopic sore, inflammation area, ulcer score, colon width and length. GPER activation by a selective agonist G-1, an endogenous agonist 17β-estradiol and a selective nuclear estrogen receptor down-regulator ICI 182.780 results in macroscopic and ulcer score improvement, decline of inflammation area and colon width. ICI 182,780 being a pure anti-estrogen capable of preventing the dimerization of nuclear estrogen receptors and leading to their degradation, acts as a GPER agonist^21^. Our analysis showed that ICI 182.780 effectively reduces colitis similarly to G-1 or 17β-estradiol suggesting that primarily GPER but not nuclear estrogen receptor mediates anti-inflammatory action of estrogens. Additionally, G-1 as opposed to G-15 was able to invert shortening of colon, which was caused by TNBS. Histological analysis confirmed immunomodulatory function of GPER in murine model of CD. The decrease of the local CRP protein content in TNBS group treated with G-1 in comparison to the TNBS group is another proof of the reduction of inflammation. CRP is a highly sensitive, acute-phase protein response to immune reaction after tissues injury, damage or infection. Significant up-regulation of CRP protein level was observed in the case of TNBS group and G-15-treated group. Some evidence points to a correlation between high-sensitivity CRP concentration at diagnosis and the risk of surgery in CD patients^22^. Our survival ratio analysis showed that G-1 and ICI 182.780 are able to reduce mortality of mice with TNBS-induced CD. On the other hand, we observed that 17β-estradiol treatment did not improve survival ratio in murine model of CD. This phenomenon may be related to the fact that 17β-estradiol is agonist not only for GPER but also for nuclear estrogen receptors and lack of mortality changes in 17β-estradiol treated group in relation to TNBS group may be associated with competition of estrogen receptors for 17β-estradiol binding^21^. Additionally, we have demonstrated that GPER activation by selective agonists G-1 and ICI 182.780 induces changes in nuclear estrogen receptors expression profile which are completely different from changes induced by 17β-estradiol in murine model of CD, that is no doubt important for estrogen signaling and can be the cause of differences observed in survival ratio analysis.

Decrease of inflammation in murine model of CD seems to be directly related with decline of GPER expression at both mRNA and protein level after G-1 and 17β-estradiol treatment. Interestingly, we have also demonstrated some effects of GPER activation/inhibition on ERα and ERβ expression, suggesting that GPER may interact with nuclear estrogen receptors in the modulation of colon inflammation. Several studies emphasize that estrogen signaling network is strictly synchronized by cross-talk between estrogen receptors ERα and ERβ and GPER^23^. In TNBS-induced colitis GPER activation was accompanied by down-regulation of ERβ in G-1 and 17β-estradiol treated groups at the mRNA and protein level, respectively. Interestingly, we did not observed any changes in ERβ protein content in the case of ICI 182.780 treatment.

GPER has been characterized as membrane-bound estrogen receptor as opposed to ERα and ERβ, which are able to translocate to the nucleus. Our immunohistochemistry analysis revealed cytoplasmic localization of GPER in enterocytes and goblet cell membranes in TNBS and GPER agonists-treated groups. GPER antagonist treatment was associated with nuclei and cytoplasm localization of GPER. Cytoplasmic localization of GPER in mast cells of lamina propria in mucosa obtained from healthy subjects and irritable bowel syndrome patients was documented by Qin *et al*.^24^. Additionally, GPER presence in myenteric neurons in the female mouse intestine at the mRNA and protein level was suggested^25^. In turn, murine intestine cross-section staining revealed localization of GPER not only in myenteric neurons but also in non-neuronal cells population^26^. Interestingly, we have shown that GPER activation was related with ERα accumulation in cytoplasm of goblet cells, while GPER inhibition led to ERα translocation to nucleus, suggesting that GPER and ERα can cooperate to promote pro-inflammatory reaction in CD. In the case of ERβ we observed cytoplasmic localization in the apical and subapical domain of the crypts and epithelium. This phenomenon may be related to the fact that ERβ may be involved in cell-cell and tight junction protein interactions, especially that we observed strong ERβ staining in the apical domain of the crypts and epithelium in TNBS group. Co-staining of estrogen receptors in cross-section obtained from murine model of CD and treated groups disclosed lack of GPER, ERα or/and ERβ co-localization.

To determine the effects of GPER activation at the molecular level a panel of genes/proteins involved in cell signaling and immune response was examined. Our study on murine model of CD clearly showed that GPER activation mediates ERK but not AKT signaling pathway. In TNBS-induced colitis G-1 treatment significantly reduced ERK 1/2 and activated ERK 1/2 protein level. In line, co-localization immunofluorescence study confirmed stronger GPER and phosphoERK 1/2 staining in G-1 treated group. Interestingly, we demonstrated that the entire ERK pathway is down-regulated. The decrease of MEK 1 and MEK 2 protein level after GPER activation by G-1 in murine model of CD was observed. Ultimately, GPER-mediated decrease of ERK activity have consequence for down-regulation of some target genes. We observed decline of the copy number of cyclin D1 mRNA in G-1-treated group in relation to TNBS group indicates the possible involvement of GPER in cell cycle regulation (see Supplementary Fig. S8). Immunohistochemical studies have shown higher expression of cyclin D1 and cyclin E in cross-section obtained from CD patients compared to healthy control^27^. Since cyclin D1 expression is regulated by mitogenic signals^28^ it can be assumed that GPER may mediate cyclin D1 expression at mRNA level *via* ERK pathway in murine model of CD.

Accumulating evidence and data presented by us indicated that GPER affects expression of estrogen receptors ERα and ERβ, which are capable to many target genes regulation. Therefore, the importance of GPER activation/inhibition for estrogen receptors ERα and ERβ and estrogen-related receptors ESRRα, ESRRβ and ESRRγ seems to be crucial for a better understanding of estrogen signaling in colon. It has been shown that estrogen-related receptors do not bind or respond to endogenous estrogens. However, it is suggested that ESRR may cooperate with nuclear estrogen receptors. *In vitro* experiments demonstrated that ESRR may bind to nuclear estrogen receptors biding site in ER-negative cell lines^29^. We demonstrated GPER-dependent changes in estrogen-related receptors expression in murine model of CD. GPER agonist treatment was found to be associated with down-regulation of Esrrα and Esrrγ transcript level, while no differences in Esrrβ in all treatment group compared to TNBS group was observed. These data suggest that not only nuclear estrogen receptors but also GPER may cooperate with estrogen-related receptors. However, since ICI 182.780 is an agonist for GPER but also acts as an antagonist of nuclear estrogen receptor it is not clear whether our observations are the result of GPER activation or nuclear estrogen receptor inhibition or cross-talk between estrogen receptors.

There is no doubt that STAT3 signaling is activated by number of cytokines such as IL-6, IL-10 or IL-23 and is able to modulate anti-apoptotic, proliferation and angiogenic target genes^30^. In response to the inflammation, STAT3 is able to multiple downstream target genes modulation, including many pro-inflammatory cytokines. STAT3 signaling pathway acts as a pro-inflammatory positive loop and STAT3 up-regulation and activation consequently leads to maintenance of inflammation. As it was shown knockout mice with diethylnitrosoamine induced hepatocarcinogenesis are characterized by higher level of pro-inflammatory cytokines IL-6, IL-1β and TNFα in relation to wild type animals^31^. Decline of Stat3 mRNA copy number in murine model of CD after GPER activation by G-1 was noted. Immune reaction may be modulated also by NFκB signaling in response to pro-inflammatory cytokines or growth factors. Our gene expression study revealed that GPER activation led to down-regulation of Rela mRNA expression, crucial subunit of NFκB complex which is able to induced survival, proliferation and inflammation-related target genes. Another genes involved in inflammation, which enhance the synthesis of pro-inflammatory mediators such as IL-6 and IL-8 are genes coding for NOS2 and COX-2^32–34^. Decline in mRNA expression of Nos2 after G-1 treatment in TNBS-induced colitis was noted. However, ICI 182.780 administration did not cause the same effect like G-1 administration, suggesting that nuclear estrogen receptors have also impact on NOS2 regulation in CD. On the other hand, both G-1 and ICI 182.780 administration was found to be related with down-regulation of Cox-2 mRNA expression in murine model of CD. Lower expression of Vegfa mRNA in the case of G-1 treatment in murine model of CD was also shown. VEGF is crucial regulator of angiogenesis, which may be modulated by inflammation and is associated not only with progression and tumor microenvironment but also seems play to significant role in autoimmune diseases. Immunohistochemical analysis revealed significantly higher VEGF expression in both IBD type i.e., CD and UC compared to control group^35^. In line, higher serum level of VEGF in patients with active CD than in patients with remission was indicated^36^. Additionally, Bustos *et al*. proved that GPER is able to mediate changes in HIF1-α and VEGFA expression in colorectal cancer cells under normoxia and hypoxia condition which was associated with CRC development^37^. It is suggested that a crucial role in the pathology of colitis plays transit receptor potential vanilloid 4. TRPV4 is overexpressed in CD and exert pro-inflammatory action in colon^38,39^. In TNBS-induced murine model of CD, Fichna *et al*. observed an improvement of macroscopic, ulcer and macroscopic score as well as a decrease of MPO activity after TRPV4 agonist administration^38^. In our study, we demonstrated that GPER activation by specific GPER agonist G-1 is associated with a decrease of Trpv4 mRNA expression, while activation by endogenous agonist of estrogen receptors, i.e.17β-estradiol leads to overexpression of Trpv4 copy number. Our results confirm that GPER acts differentially than nuclear estrogen receptors which are able to mediate some pro-inflammatory effects in CD.

One of the mechanisms of post-transcriptional regulation affecting gene expression are microRNAs. Small non-coding RNAs are able to target gene silencing and proved to be important pro-inflammatory gene modulators. Our analysis of selected microRNA revealed that GPER activation induced changes of miR-145, miR-148-5p and miR-592 expression in TNBS mice. In murine model of CD supplemented with GPER agonists the decrease of miR-145 and miR-148-5p was stated. Experimental data indicate that miR-145 regulates immune reaction and NFκB signaling pathway in atherosclerosis. *In vitro* and *in vivo* studies confirmed relationship between overexpression of miR-145 and phosphorylation of IKBa and STAT3 as well as acetylation of p65^40^. Additionally, pivotal role of miR-143/145 family in intestinal regeneration was proven^41^. Also miR-148/152 family of prognostic potential in various cancer types seems to play a role in immune response. It is suggested that miR-148a is an indirect tumor suppressor that modulates colitis and colitis-associated tumorigenesis by suppressing the signaling by NFκB and STAT3^42,43^. On the other hand, it was found that miR-148a can be regulated *via* estrogen receptors, i.e. GPER and ERα^44,45^. In turn, expression changes observed in the case of miR-592 seem to be directly related with a decrease of nuclear estrogen receptors level. Overexpression of miR-592 in murine model of CD treated with ICI 182.780 was noted. Importance of miR-592 was extensively examined in the context of cancer cells but there are conflicting data concerning miR-592 and cancer development. It was shown that miR-592 is down-regulated in human glioma cell lines and tissue specimens while up-regulated in colorectal cancer in relation to control group^46,47^. Nevertheless, based on our results from murine experiments and in view of anti-inflammatory action of ICI 182.780, nuclear estrogen receptors degradation and accompanying overexpression of miR-592 may play a role in colitis prevention.

## Conclusions

This study has shown that GPER agonists mediate immunosuppressive action of GPER in Crohn’s disease and that GPER-targeting therapy may be promising approach in maintaining remission in Crohn’s disease. Crohn’s disease is characterized by alternating occurrence of active stage with inflammation causing colon damage and remission phase of disease. In the therapy of Crohn’s disease patients, maintaining remission of disease during colon return to the stage of homeostasis is the crucial event. We demonstrated that GPER activation leads to improvement of clinical and endoscopic image of CD in mice. Our analysis revealed that GPER is able to mediate anti-inflammatory effects observed not only at the macro- and microscopic level but also at the molecular level leading to ERK signaling inhibition and decline of the expression of immune response genes. It seems to be essential not only for Crohn’s disease but also for colorectal cancer development related with inflammation for which inflammatory bowel diseases are one of the significant risk factor.

## Materials and Methods

### Human study group and colon mucosa sample collection

In total 21 men with CD (age 29–86) and 27 male unrelated controls (age 32–83) were enrolled to the study. CD was evaluated based on clinical, radiological, endoscopic and histological criteria recommended by European Crohn’s and Colitis Organization. Colonic forceps biopsies were taken from patients hospitalized at the Department of Digestive Tract Disease, Faculty of Medicine at the Medical University of Lodz, Poland and kept at −80 °C for further analysis. The study was conducted in accordance with the ethical principles of the 1975 Declaration of Helsinki and the Committee of Bioethics of Medical University of Lodz and the Committee of Bioethics of University of Lodz approved the study protocols (RNN/93/12KE and 21/KBBN-UŁ/II/2015, respectively). All participating subject gave written, informed consent prior to enrollment.

### Animals

Male BALB/c mice were obtained from the Animal Facility of the University of Lodz, Poland. All animals used in experiments weighed 25–28 g. The animals were housed at constant temperature (22–24 °C), relative humidity 55 ± 5% and maintained under 12 h light/dark cycle (lights turned on 6 a.m.) with a free access to standard chow pellets and tap water *ad libitum*. To minimize circadian influence, all experiments were performed between 8:00 a.m. and 12:00 a.m. after 7 days of acclimatization. The experimental protocol was approved by the Local Ethical Committee at the Medical University of Lodz (28/ŁB29/2016) and complied with the European Communities Council Directive of September 22, 2010 (2010/63/EU). The study was carried out in strict accordance with the institutional recommendations. Every effort was taken to minimize animal suffering and to reduce the number of animals used. Groups of 10–15 animals were used in all *in vivo* experiments.

### Murine model of Crohn’s disease and treatments

Semi-chronic colitis was induced by intracolonic administration of 2, 4, 6-trinitrobenzenesulfonic acid (TNBS) at day 1. Briefly, mice were anesthetized with 1% isoflurane (Baxter Healthcare Corp., USA) and TNBS (4 mg in 0.1 ml of 30% ethanol in 0.9% NaCl) was instilled into the distal colon through a catheter (3 cm proximally into the anus). Control mice received vehicle alone (0.1 ml of 30% ethanol in 0.9% NaCl). Mice were monitored daily for clinical parameters including body weight, rectal bleeding, stool consistency and mortality. Starting at day 4 mice were treated with GPER ligands. An appropriate concentrations of studied ligands, i.e. G-1, G-15 (Tocris Bioscience, United Kingdom), 17β-estradiol and ICI 182.780 (Sigma Aldrich, Germany) were evaluated before final experiment on animal model based on *in vitro* experiments. GPER agonist G-1, 17β-estradiol and ICI 182.780 (at the dose of 20 mg/kg) or antagonist G-15 (at the dose of 5 mg/kg) in the final volume of 100 μl were injected intravenously once daily. All drugs were dissolved in 5% dimethyl sulfoxide (DMSO) in 0.9% NaCl. Control animals received equal volume of 5% DMSO in 0.9% NaCl as a vehicle. To determine the activity of the compounds used, different routes of administration (intraperitoneal and intravenous) and different doses (1, 5, 10 and 20 mg/kg) were tested in mice and the most efficient were selected for further studies. On day 8 animals were sacrificed by cervical dislocation and evaluation of macroscopic score was performed. Colon samples were collected and kept at −80 °C for further analysis. Colon samples for histology and immunohistochemistry were kept in 4% paraformaldehyde (Sigma Aldrich, Germany) for 24 h at 4 °C.

### Macroscopic score evaluation

The colon and caecum were gently removed, washed with phosphate-buffered saline (PBS) and immediately examined. Macroscopic colonic damage was assessed by a well-established semi-quantitative scoring system^48^. The total macroscopic score is a sum of the following parameters: for ulcer score 0.5 points for each 0.5 cm, the wall thickness measured in mm (thickness of *n* mm corresponds to *n* scoring points), the presence of haemorrhage, faecal blood and diarrhea increased the score by 1 point for each additional feature and presence of adhesion (0–2).

### Histology and microscopic score evaluation

Colon samples were dehydrated, embedded in paraffin, sectioned at 5 μm using Leica RM2255 fully automated rotary microtome (Leica-Microsystems, Germany) and mounted onto slides. The section were stained with hematoxylin and eosin (Sigma Aldrich, Germany) and examined. Photographs were taken using hardware (AX-70 light microscope, Imaging Solutions Camera) and software (Cell F Imaging Software) from Olympus and Olympus Soft Imaging Solutions (Germany), respectively. Microscopic total damage score was determined based on the presence (score = 1) or absence (score = 0) of goblet cell depletion and crypt abscesses, the destruction of the mucosa architecture (normal = 1, moderate = 2, extensive = 3), the extent of muscle thickening (normal = 1, moderate = 2, extensive = 3), and the presence and degree of cellular infiltration (normal = 1, moderate = 2, transmural = 3).

### Immunohistochemistry analysis

For immunohistochemistry analysis, formalin-fixed paraffin-embedded sections (5 µm) were used. Sections were deparaffinized and dehydrated and subsequently were incubated in boiling sodium citrate buffer (10 mM sodium citrate; 0.05% Tween^®^20; pH = 6) for 10 minutes to antigen unmasking. To inhibit endogenous peroxidase activity 3% hydrogen peroxidase water solution was used. After 60 minutes in block solution (5% goat normal serum; 50 mM Tris/HCl; pH = 7.5; 150 mM NaCl) supplemented with 0.05% Triton X-100 the sections were incubated overnight at 4 °C with commercially available primary antibody (dilution 1:100) against GPER (ab39742), ERα (ab75635), ERβ (ab3576) or phosphoERK 1/2 (#4377). Next, the sections were washed and incubated with Alexa Fluor^®^ 488 (A-11034, Thermo Fisher Scientific, USA) or DyLight^®^ 650 (ab96886, Abcam, USA) goat anti-rabbit secondary antibodies for 60 minutes. 3,3’-dihexyloxacarbocyanine lodide (DiOC6 (3); #318426, Sigma Aldrich, Germany) diluted in TBS (50 mM Tris/HCl, pH = 7.5; 150 mM NaCl) was used to membrane staining. Afterwards, the sections was mounted with ProLong^TM^ Diamond Antifade Mountant with DAPI (P36962, Thermo Fisher Scientific, USA). For negative control, sections were incubated with only primary or secondary antibodies to determinate non-specific staining. MCF-7 cell line was used as a positive control. Leica TCS SP8 confocal microscope (Leica-Microsystems, Germany) featuring 20x and 63x/1.40 lens (HC PL APO CS2 Oil Immersion) was used for microscopic imaging. Supercontinuum laser with 488 nm excitations and emission at 480–520 nm for Alexa Fluor^®^ 488 and with 654 nm excitations and emission at 660–680 nm for DyLight^®^ 650 was used. For visualization of membranes staining with DiOC6 (3) supercontinuum laser with 485 nm excitation and emission at 538–595 nm was applied. For nuclei stained with DAPI for excitation and emission parameters were 405 nm and 460–480 nm, respectively. Confocal microscopy analysis was performed in the Laboratory of Microscopic Imaging and Specialized Biological Techniques at the Faculty of Biology and Environmental Protection, University of Lodz.

### Level of myeloperoxidase activity

The colon sections were isolated, washed in PBS and homogenized in hexadecyltrimethylammonium bromide (HTAB) buffer (0.5% HTAB in 50 mM potassium phosphate buffer pH = 6.0) using Ika Ultra Turrax Disperser T25 Digital 2 (Sigma Aldrich, Germany). The homogenates were centrifuged at 13 200 × g for 15 min and the supernatants were transferred to new tubes. 7 µl of supernatant were added on a 96-well plate, followed by 200 µl of 50 mM potassium phosphate buffer (pH = 6.0), containing 0.167 mg/ml of O-dianisidine hydrochloride and 0.05 µl of 1% H_2_O_2_. Absorbance was measured on iMARK Microplate Reader (Bio-Rad, UK) at 450 nm after 30 and 60 seconds. All measurements were performed in triplicates.

### RNA and microRNA isolation

Total RNA isolation were performed using commercially available TRIsure^TM^ (Bioline, Australia) and microRNA Concentrator (A&A Biotechnology, Poland). Colon samples were minced and homogenized in TRIsure^TM^. After centrifugation and phases separation, aqueous phase was mixed 3:1 (v/v) with isopropanol and loaded on the column. Subsequent steps were conducted according to manufacturer’s protocol. The quality and quantity of RNA and microRNA were estimated spectrophotometrically with BioPhotometer Plus (Eppendorf, Germany). The RNA and microRNA were characterized with A_260_/A_280_ ratio, which was in the range of 1.70–2.00.

### Reverse transcription and quantitative real-time PCR

cDNA and microRNA synthesis was performed with High-Capacity cDNA Reverse Transcription Kit or TaqMan^®^ microRNA Reverse Transcription Kit (Applied Biosystems, USA) in accordance with the manufacturer’s protocol. Total RNA and microRNA (1 μg or 350 ng, respectively) were used in reverse transcription reaction with the following incubation steps: 25 °C for 10 minutes, 37 °C for 120 minutes and 85 °C for 5 minutes for RNA and 16 °C for 30 minutes, 42 °C for 30 minutes and 85 °C for 5 minutes for microRNA. Quantification of mRNA and microRNA was performed using the real-time PCR method with FAM dye-labeled TaqMan^®^ probes (Applied Biosystems, USA). The reaction mixture consisted of cDNA, TaqMan^®^ Master Mix II, no UNG, TaqMan^®^ Assays (see Supplementary Table S1 and S2) and RNase-free water in total volume of 10 μl. Cycle parameters for TaqMan^®^ Assays were as follows: initial denaturation at 95 °C for 10 minutes, followed by 40 cycles of sequential incubations at 95 °C for 15 seconds and at 60 °C for 1 minutes. Obtained results were normalized to the expression of GAPDH (glyceraldehyde 3-phosphate dehydrogenase) for studied genes and miR-26b for exanimated microRNA. All experiments were performed as triplicates. The real-time PCR was performed using in Master Realplex4s (Eppendorf, Germany). The fluorescent dye emission was a function of the cycle number. The initial amount of the temple was evaluated as a Ct parameter. Ct value corresponded to the threshold cycle number at witch PCR amplification reached a significant threshold. The relative expression level was calculated as 2^−∆Ct^ × 1000. The results expressed as number of examined mRNA or microRNA copies per 1000 copies of mRNA for GAPDH or miR-26b, respectively.

### Western blot analysis

Proteins were isolated in the RIPA buffer (50 mM Tris/HCl, pH = 7.6; 150 mM NaCl; 1% Triton X-100; 0.1% SDS; 1% sodium deoxycholate; 2 mM EDTA) supplemented with 1 mM phenylmethane sulfonyl fluoride (PMSF) using ultrasonic homogenizer (Sonic&Meterials, USA). The homogenates were cleared by centrifugation at 5 000 rpm for 10 minutes. Total protein concentration was evaluated in each samples in triplicate using Lowry protocol. 25 μg of protein samples were separated on 8% or 12% polyacrylamide gels in electrophoresis buffer (25 mM Tris, pH = 8.3; 192 mM glycine; 0.1% SDS) and electrotransferred in transfer buffer (25 mM Tris, pH = 8.3; 192 glycine; 20% methanol) using wet system onto polyvinyl difluoride (PVDF) membranes (pore size 0.45 µm, Thermo Fisher Scientific, USA). Membranes were blocked 60 minutes in 5% casein before incubation with commercially available primary antibodies (dilution 1:100–1:1000) against CRP (sc-69770), GPER (ab39742), ERα (ab75635), ERβ (ab3576), ERK 1/2 (#9101), phosphoERK 1/2 (#4377), MEK1 (sc-6250), MEK2 (sc-13159), AKT (#9272) or phosphoAKT (#9271) overnight at 4 °C. The optimal dilution of antibodies was selected before the final experiment. For each sample a separate analysis was performed using β-actin antibodies conjugated with horseradish peroxidase (HRP) (dilution 1:2000, sc-47778). Primary antibodies were purchased in Santa Cruz Biotechnology, USA and Abcam, USA and Cell Signaling Technology, United Kingdom. Subsequently, membranes were incubated with secondary antibodies (dilution 1:5000) coupled with HRP (Thermo Fisher Scientific, USA). Immunoreaction was visualized using Clarity^TM^ Western ELC Substrate (Bio-Rad, USA) and X-ray films (Fujifilm, Japan). The intensities of the visualized signals were analyzed densitometrcially by using Gel Pro Analyzer v3.0 for Windows (Media Cybernetics, USA).

### Statistical analysis

Statistical analysis was performed using GraphPad Prism 5.0 (GraphPad Softwere Inc., USA). All data are presented as means ± standard error of mean (SEM). Non-parametric Mann-Whitney U test and ANOVA followed by Newman-Keuls post-hoc test were used for comparison of studied groups. *P*-values < 0.05 was considered statistically significant.

## Supplementary information

Supplementary information

## Data Availability

The datasets generated and/or analyzed during the current study are available from the corresponding author on reasonable request.
