# Supplementary material for: G protein-coupled estrogen receptor mediates anti-inflammatory action in Crohn’s disease
Source: Sci Rep. 2019 May 1;9:6749. doi: 10.1038/s41598-019-43233-3 (PMC6494840; doi:10.1038/s41598-019-43233-3)
Supplement: Supplementary file 1 — Supplementary information [file 41598_2019_43233_MOESM1_ESM.pdf]

Supplementary information

G protein-coupled estrogen receptor mediates anti-inflammatory action in Crohn's disease

Damian Jacenik[a]*, Marta Zielińska[b], Anna Mokrowiecka[c], Sylwia Michlewska[d,e],
Ewa Małecka-Panas[c], Radzisław Kordek[f], Jakub Fichna[b], Wanda M. Krajewska[a]*

[a] – Department of Cytobiochemistry, Faculty of Biology and Environmental Protection, University of Lodz, Pomorska St. 141/143, 90-236 Lodz, Poland

[b] – Department of Biochemistry, Faculty of Medicine, Medical University of Lodz, Mazowiecka St. 6/8, 92-215 Lodz, Poland

[c] – Department of Digestive Tract Diseases, Faculty of Medicine, Medical University of Lodz, Stefana Kopcinskiego St. 22, 90-001 Lodz, Poland

[d] – Department of General Biophysics, Faculty of Biology and Environmental Protection, University of Lodz, Pomorska St. 141/143, 90-236 Lodz, Poland

[e] – Laboratory of Microscopic Imaging and Specialized Biological Techniques, Faculty of Biology and Environmental Protection, University of Lodz, Banacha St. 12/16, 90-237 Lodz, Poland

[f] – Department of Pathology, Chair of Oncology, Medical University of Lodz, Pomorska St. 521, 92-213 Lodz, Poland

* – corresponding author: Damian Jacenik, Department of Cytobiochemistry, Faculty of Biology and Environmental Protection, University of Lodz, Pomorska St. 141/143, 90-236 Lodz, Poland, tel: +48 42 635 52 99, email: damian.jacenik@biol.uni.lodz.pl

* – corresponding author: Wanda M. Krajewska, Department of Cytobiochemistry, Faculty of Biology and Environmental Protection, University of Lodz, Pomorska St. 141/143, 90-236 Lodz, Poland, tel: +48 42 635 44 87, email: wanda.krajewska@biol.uni.lodz.pl

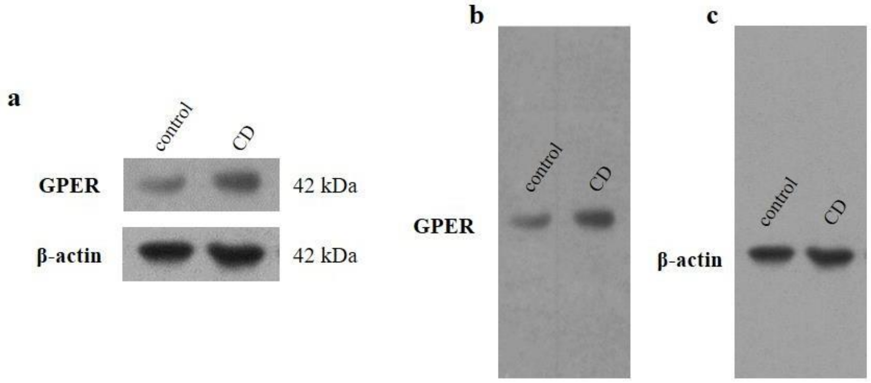

Supplementary Figure S1. Representative main figure (a) and representative image of full-length immunoblot from GPER (b) and β-actin (b) protein expression analysis in colon section obtained from control and CD patients.

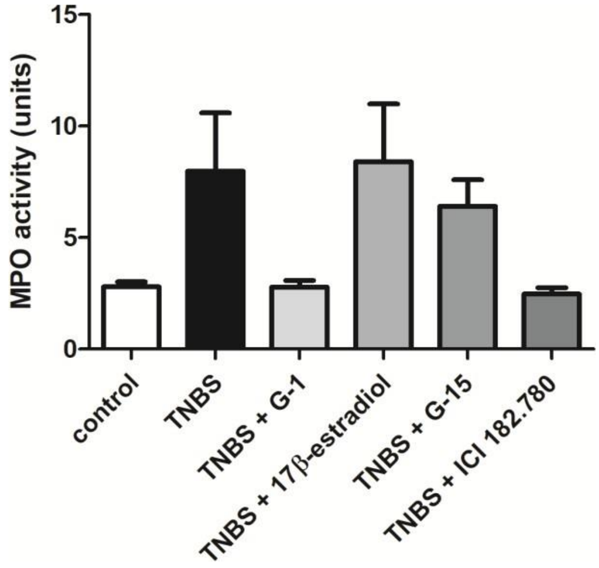

Supplementary Figure S2. MPO activity in colon section obtained from control, TNBS and treated groups. Data are presented as means ± SEM. 10 – 15 mice per group.

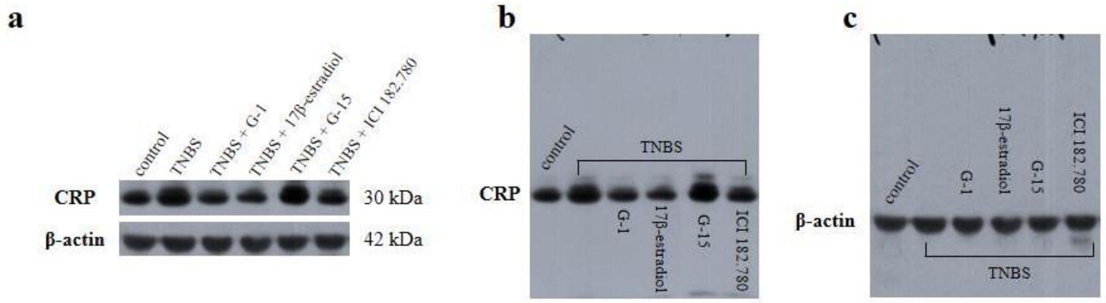

Supplementary Figure S3. Representative main figure (a) and representative image of full-length immunoblot from CRP (b) and β-actin (c) protein expression analysis in colon section obtained from control, TNBS and treated groups. 10 – 15 mice per group.

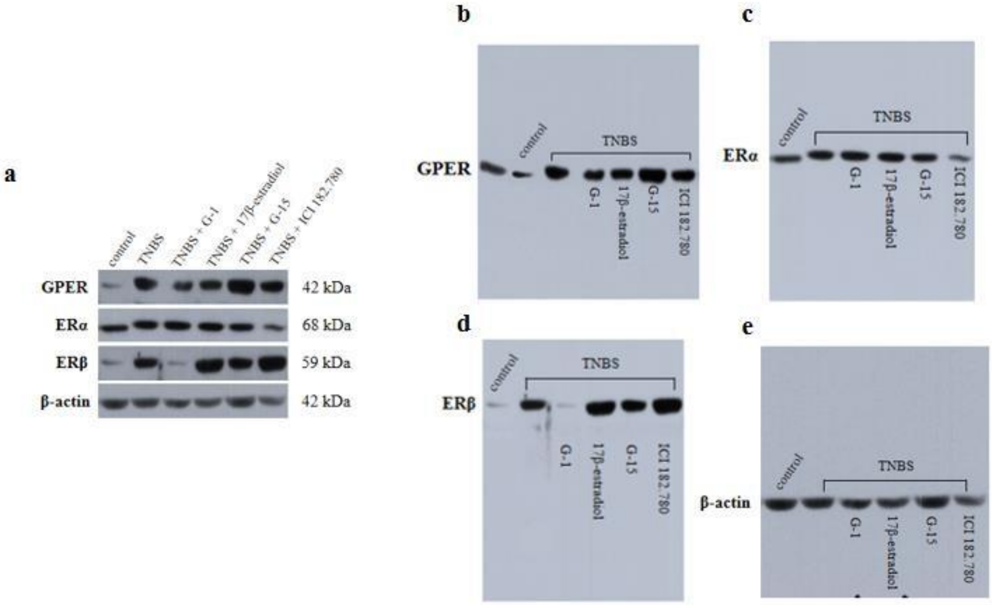

Supplementary Figure S4. Representative main figure (a) and representative image of full-length immunoblot from GPER (b), ERα (c), ERβ (d) and β-actin (e) protein expression analysis in colon section obtained from control, TNBS and treated groups. 10 – 15 mice per group.

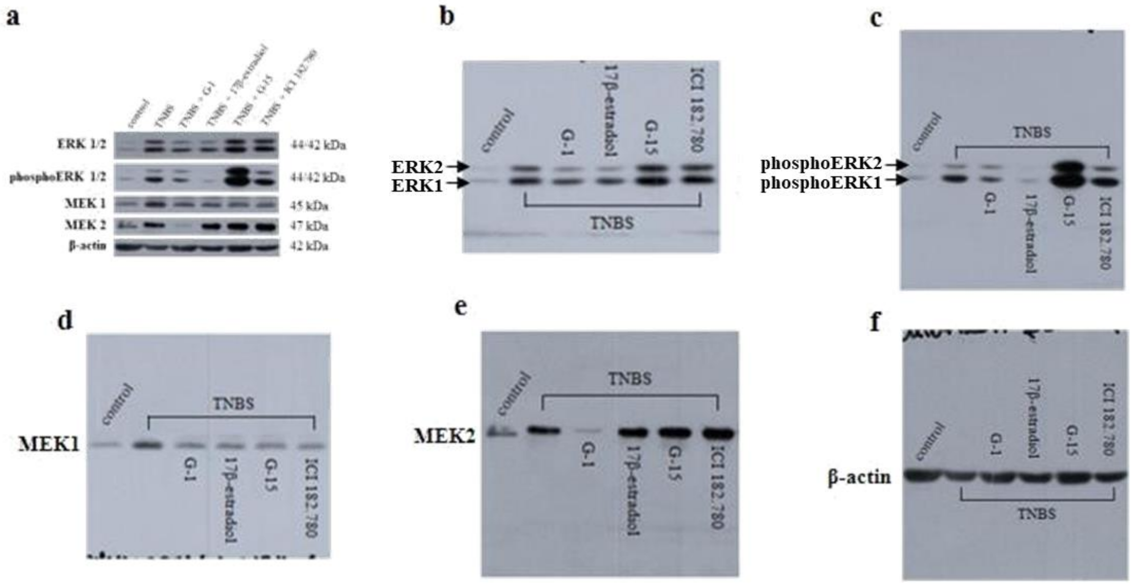

Supplementary Figure S5. Representative main figure (a) and representative image of full-length immunoblot from ERK1/2 (b), phosphoERK1/2 (c), MEK1 (d), MEK2 (e) and β-actin (f) protein expression analysis in colon section obtained from control, TNBS and treated groups. 10 – 15 mice per group

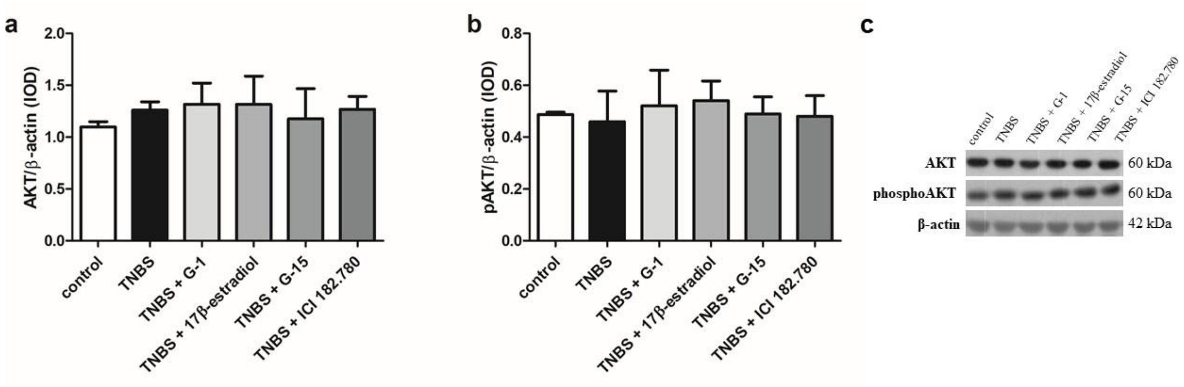

Supplementary Figure S6. AKT (a) and phosphoAKT (b) protein level and representative immunoblot of AKT and phosphoAKT protein analysis (c) in colon section obtained from control, TNBS and treated groups. 10 – 15 mice per group.

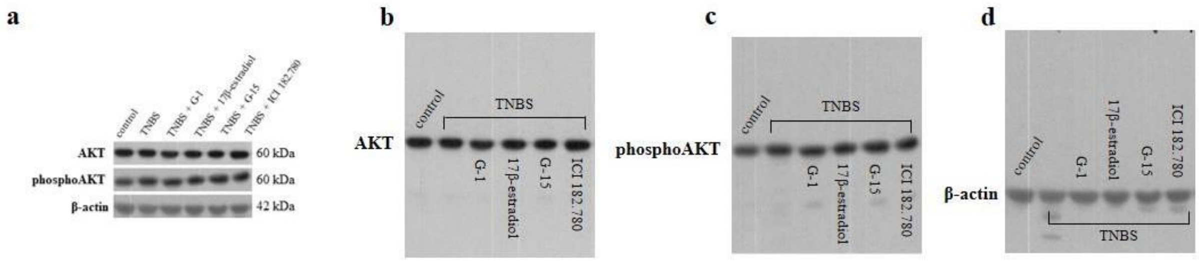

Supplementary Figure S7. Representative main figure (a) and representative image of full-length immunoblot from AKT (b), phosphoAKT (c) and β-actin (d) protein expression analysis in colon section obtained from control, TNBS and treated groups. 10 – 15 mice per group.

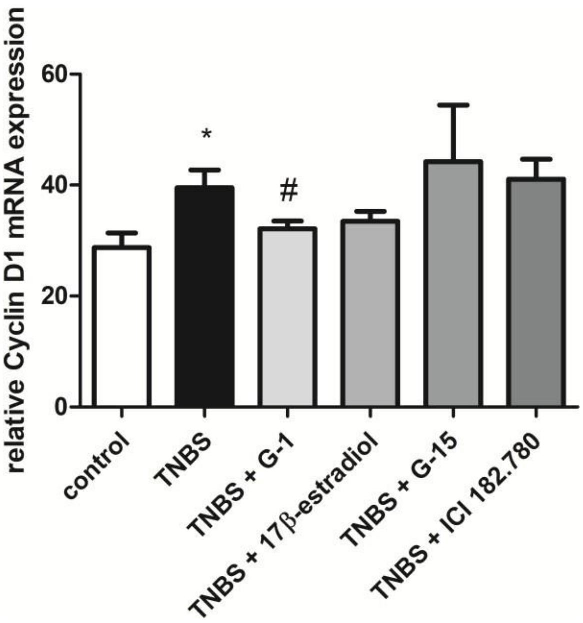

Supplementary Figure S8. Cyclin D1 expression at the mRNA level in colon section obtained from control, TNBS and treated groups. Data are presented as means ± SEM; *P < 0.05 vs. control; #P < 0.05 vs. TNBS. 10 – 15 mice per group.

Supplementary Table S1. TaqMan® gene expression assays ID used in real-time PCR analysis and their amplicon length.

Species	Gene	Assay ID	Amplicon length
**Human**	*GPER*	Hs00173506_m1	58
	GAPDH	Hs99999905_m1	122
**Mouse**	*Gper*	Mm01194815_m1	53
	Ccnd1	Mm00432359_m1	58
	Cox-2	Mm00478374_m1	80
	Esr1	Mm00433149_m1	56
	Esr2	Mm00599821_m1	69
	Esrrα	Mm00433143_m1	133
	Esrrβ	Mm00442411_m1	55
	Esrrγ	Mm01314576_m1	61
	Nos2	Mm00440502_m1	66
	Rela	Mm00501346_m1	67
	Stat3	Mm00456968_m1	120
	Trpv4	Mm00499025_m1	56
	Vegfa	Mm00437306_m1	61
	Gapdh	Mm99999915_g1	109

Supplementary Table S2. Table 2. TaqMan® microRNA assays ID and their sequence used in real-time PCR analysis.

Gene	Sequence	Assay ID
miR-145	GUCCAGUUUUCCCAGGAAUCCCU	002278
miR-148-5p	AAAGUUCUGAGACACUCCGACU	002134
miR-592	UUGUGUCAAUAUGCGAUGAUGU	001546
miR-26b	UUCAAGUAAUUCAGGAUAGGU	000407
